# Membrane Labeling of Coral Gastrodermal Cells by Biotinylation: The Proteomic Identification of Surface Proteins Involving Cnidaria-Dinoflagellate Endosymbiosis

**DOI:** 10.1371/journal.pone.0085119

**Published:** 2014-01-07

**Authors:** Hsing-Hui Li, Zi-Yu Huang, Shih-Png Ye, Chi-Yu Lu, Pai-Chiao Cheng, Shu-Hwa Chen, Chii-Shiarng Chen

**Affiliations:** 1 Graduate Institute of Marine Biotechnology, National Dong Hwa University, Pingtung, Taiwan; 2 Taiwan Coral Research Center, National Museum of Marine Biology and Aquarium, Pingtung, Taiwan; 3 Department of Biochemistry, College of Medicine, Kaohsiung Medical University, Kaohsiung, Taiwan; 4 Department of Biochemistry and Molecular Biology, College of Medicine, National Cheng Kung University, Tainan, Taiwan; 5 Institute of Information Science, Academia Sinica, Taipei, Taiwan; 6 Department of Marine Biotechnology and Resources, National Sun Yat-Sen University, Kaohsiung, Taiwan; Mount Allison University, Canada

## Abstract

The cellular and molecular-scale processes underlying the stability of coral-*Symbiodinium* endosymbioses remain unclear despite decades of investigation. As the coral gastroderm is the only tissue layer characterized by this unique symbiotic association, the membranes of these symbiotic gastrodermal cells (SGCs) may play important roles in the initiation and maintenance of the endosymbiosis. In order to elucidate the interactions between the endosymbiotic dinoflagellates and their coral hosts, a thorough characterization of SGC membranes is therefore required. Cell surface proteins of isolated SGCs were biotinylated herein by a cell impermeant agent, biotin-XX sulfosuccinimidyl ester. The *in situ* distribution of these biotinylated proteins was uncovered by both fluorescence and transmission electron microscopic imaging of proteins bound to Alexa Fluor® 488-conjugated streptavidin. The identity of these proteins was then determined by two-dimensional gel electrophoresis followed by liquid chromatography-tandem mass spectrometry. Nineteen (19) proteins were identified, and they are known to be involved in the molecular chaperone/stress response, cytoskeletal remodeling, and energy metabolism. These results not only reveal the molecular characters of the host SGC membrane, but also provide critical insight into understanding the possible role of host membranes in this ecologically important endosymbiotic association.

## Introduction

The coral-*Symbiodinium* endosymbiosis is a unique phenomenon in which a phototrophic dinoflagellate (i.e., the endosymbiont) lives within the gastrodermal cell of the coral host [1], [2]. This endosymbiosis is responsible for the construction of coral reefs across Earth’s tropical seas [1], though the processes involved in its regulation are poorly understood. Cell biology approaches have attempted to elucidate four processes that are integral to the biology of these associations: (i) recognition [2], [3] and phagocytosis [4], [5] of *Symbiodinium* into host symbiotic gastrodermal cells (SGCs); (ii) regulation of host cell growth and proliferation of the endosymbionts; (iii) metabolic exchanges and the nutrient dialogue between *Symbiodinium* and their host cells; and (iv) host coral calcification [6], [7].

After the phagocytosis of the *Symbiodinium* into the host gastrodermal cells, a symbiosome membrane is enveloped around the endosymbionts [8], [9], [10]. Although the steps involved in symbiosome membrane formation remain unclear, immunofluorescence analyses have indicated that there are outer and inner layers, which originate from the host and endosymbiont, respectively [8]. Furthermore, 17 symbiosome membrane-associated proteins have been identified, and they include membrane receptors involved in cell recognition, as well as proteins involved in cytoskeletal remodeling, ATP synthesis/proton homeostasis, transport, the stress response, and prevention of apoptosis [9].

Past studies have shown that there is active membrane trafficking of the plasma membrane of SGCs of the reef-building coral *Euphyllia glabrescens* [11]. It was furthermore shown that the degree of *Symbiodinium* photoinhibition is related to perturbation of SGC membrane trafficking and metabolism. The SGC plasma membranes may also play pivotal roles in the recognition and phagocytosis of *Symbiodinium* during the initial steps of the endosymbiotic process [11], [12]. As such, SGC membranes may act to regulate the stability of the association between the host coral and its intracellular dinoflagellates. However, the composition of SGC plasma membranes, including their proteins and lipids constituents, remains unclear. To greater understand the cellular mechanisms underlying stable cnidarian-dinoflagellate endosymbioses, a more thorough investigation of the surface proteins of SGCs is therefore essential. This study aimed to identify surface proteins of SGCs in order to elucidate the molecular characteristics of the host plasma membrane and provide insight into the possible role of these proteins in regulation of this endosymbiotic association.

## Materials and Methods

### 1. Reagents and Culture Media

All chemicals were of analytical grade. Iscove’s modified Dulbecco’s medium (IMDM, pH 7.4) (Gibco®, Invitrogen, Carlsbad, CA, USA) was prepared with 0.3024% NaHCO_3_ and 10% fetal bovine serum. Filtered seawater (FSW) was generated by filtering seawater through a Stericup® filter unit (0.22 µm pore size; Merck Millipore, Billerica, MA, USA). Artificial seawater (ASW) was prepared in HEPES (10 mM) buffer (pH 8.2) and contained 420 mM NaCl, 26 mM MgSO_4_, 23 mM MgCl_2_, 9 mM KCl, 9 mM CaCl_2_, 2 mM NaHCO_3_. The osmolarity was adjusted to 1000 mOsm.

### 2. Coral Collection and Maintenance

*Euphyllia glabrescens* colonies were collected by SCUBA divers from the inlet of the Third Nuclear Power Plant (21°57.376′ N, 120°45.291′ E) at a depth of 3–8 m in Nanwan Bay, Taiwan. The coral collection was approved by the Kenting National Park Management Office. Collected colonies were transferred into seawater and placed in an upright position in a 4-ton outdoor aquarium with flow-through seawater. Colonies were maintained under a natural photoperiod with additional air circulation in the husbandry center of the National Museum of Marine Biology and Aquarium (NMMBA). A microprocessor-controlled cooler (Law-chain Computer Tech. Co., Ltd. LC-214P, Kaohsiung, Taiwan) was linked to the tank and the temperature was maintained at 26.5±1°C. Amputated tentacles were obtained from polyps of the *E. glabrescens* colonies using curved surgical scissors. These tentacles were then transferred to the laboratory and washed with FSW for further use.

### 3. Isolation of Symbiotic Gastrodermal Cells (SGCs)

SGCs were isolated from amputated tentacles according to a published procedure [13]. 5×10^5^ SGCs were suspended in 50 µL FSW and the intactness of the SGC plasma membranes were examined as previously described [13].

### 4. Biotinylation of Cell Surface Proteins for Microscopic and Proteomic Analyses

#### 4.1. Biotinylation

Approximately 1×10^7^ SGCs were first suspended in 1 mL ASW. After the addition of 10 µL biotin-XX sulfosuccinimidyl ester (Invitrogen, F-20650) stock solution (1 µg/µL, prepared in anhydrous DMSO), the cell suspension was incubated on ice for 30 min to inhibit membrane endocytosis [14]. The biotinylation reaction was terminated with 50 mM glycine at 4°C for 15 min. Cells were then pelleted (100×*g* for 5 min at 4°C) and washed with ASW. SGCs without biotinylation were used as controls.

#### 4.2. Confocal fluorescent microscopic examinations

To check whether biotinylation was successful on the SGC surfaces, 1×10^6^ biotinylated SGCs (1×10^6^ non-biotinylated SGCs were used as controls.) were suspended in 100 µL FSW. Then, 1 µL of 1 ng/µL Alexa Fluor® 488 conjugated streptavidin (Invitrogen) was added, and the mixture was incubated at room temperature (RT) for 30 min in the dark. Afterwards, the stained cells were washed with FSW and examined on a confocal microscope (Carl Zeiss, LSM510, Oberkochen, Germany).

#### 4.3. Transmission Electron Microscopy (TEM)

The biotinylated SGCs were fixed in an ice-cold fix solution of 2.5% glutaraldehyde, 2% paraformaldehyde, 0.2 M phosphate saline buffer (PBS), and 6% sucrose for 3 hr. They were then rinsed thrice with “washing buffer” (1% bovine serum albumin (BSA) and 0.1% gelatin in PBS, (pH 7.4) for 5 min. The cells were then incubated with the same washing buffer containing 30 µg/mL streptavidin conjugated with 10 nm colloidal gold (Invitrogen) for 1 hr at RT. After rinsing with washing buffer to remove unbound streptavidin, cells were post-fixed with 1% osmium tetroxide in 0.05 M phosphate buffer at 4°C for 2 hr. Cells were then washed with distilled water and pre-stained with 0.2% uranyl acetate in 70% ethanol overnight in the dark. The cells were then washed thrice with distilled water and dehydrated in a graded aqueous ethanol series (50, 70, 80, 90, 95, and 100%; 20 min at each step) at 4°C. The solvent was changed to acetone in a graded acetone/ethanol series (33%, 50%, 66%, 100% acetone; 20 min each step). Cells were then infiltrated with Spurr’s resin in acetone (33, 66, and 100% Spurr’s resin for 1 hr at each step) and embedded in gelatin capsules, which were polymerized at 70°C for 8 hrs. Afterwards, ultra-thin sections (70–80 nm) were made from the polymerized sample block and mounted on formvar-coated copper grids (300 mesh, Electron Microscopy Sciences, Hatfield, PA, USA). The specimens were developed for 4 min in silver enhancer reagent (Li silver enhancement kit, cat. number L-24919, Invitrogen) and then washed twice with deionized water for 5 minutes. After drying on filter paper for 10 min, the sections were stained with 2.5% uranyl acetate in methanol, washed with methanol, and stained with 0.4% lead citrate. After complete drying, grids were observed with a JEM-1400 transmission electron microscope (JEOL, Japan).

#### 4.4. 2D SDS-PAGE analysis of biotinylated proteins

Biotinylated SGCs were prepared as described above and suspended in 550 µL modified isotonic RadioImmunoPrecipitation Assay (RIPA) buffer (50 mM Tris, pH 7.4, 0.25% Na-deoxycholate, 150 mM NaCl, 1% NP-40, 1 mM EDTA, 1 mM Na_3_VO_4_, 1 mM NaF, 1000 mOsm.) containing a protease inhibitor cocktail (Roche, Basel, Switzerland). To this cell suspension, 1.5 g glass beads (Sigma-Aldrich, G 9268, 425–600 µm, U.S. sieve) were added, and the mixture was homogenized thrice in a TissueLyser LT (Invitrogen) containing liquid nitrogen for 5 min. Subsequently, the proteins were collected from the supernatant after centrifugation at 10,000×g at 4°C for 15 min. The dissolved salts were removed by trichloroacetic acid precipitation according to a published procedure [15], and the protein pellet was re-dissolved in rehydration solution (8 M urea, 2% CHAPS, and 20 mM DTT) for 1 hr and spun at 10,000×g at 4°C for 15 min. The concentration of soluble protein was quantified using a 2-D Quant kit (GE Healthcare, Piscataway, NJ, USA) according to the manufacturer’s recommendations.

A 13 cm DryStrip (pH 4–7) (GE Healthcare) was rehydrated in an IPGphor isoelectric focusing (IEF) system (GE Healthcare) (13 h at 50 V) with 450 µg soluble proteins mixed with 0.5% IPG buffer (pH 4–7) (GE Healthcare). IEF was performed with the following protocol: 1 h at 300 V (step), 1 h at 1000 V (gradient), 2 h at 4000 V (gradient), 1 h at 8000 V (gradient), and 4 h at 8000 V (step). Afterwards, the IPG strips were equilibrated in 1% DTT equilibration buffer (6 M urea, 2% SDS, 30% glycerol, 50 mM Tris-HCl [pH 8.8], and 0.008% bromophenol blue) for 15 min, followed by 2.5% iodoacetamide (IAA) equilibration buffer for 15 min. The equilibrated IPG strips were then placed onto a 14% polyacrylamide gel for the second-dimensional separation.

Biotinylated proteins on the 2-D SDS-PAGE gels were stained with streptavidin–Alexa Fluor® 488 (Invitrogen) and modified according to the methods described in a previous report [9], [16]. First, the gel was washed with phosphate buffered saline (PBS) for 5 min and immersed in 20 µg/ml streptavidin–Alexa Fluor® 488 for 30 min in the dark. The gel was then washed sequentially for 30 min with PBS containing 0.1% Tween-20 (thrice) and then PBS only (twice). The green fluorescent biotinylated protein spots were detected by a fluorescence image scanner (Typhoon TRIO, GE Healthcare) with an excitation wavelength of 488 nm and an emission wavelength of 526 nm. The total protein quantity of the same gel was then examined by SYPRO® Ruby gel staining according to the manufacturer’s instructions (Invitrogen). The distribution of red fluorescence protein spots was detected by the Typhoon TRIO scanner with an excitation wavelength of 532 nm and an emission wavelength of 610 nm.

#### 4.5. Identification of biotinylated proteins by LC-MS/MS analysis

The biotinylated protein spots were identified by LC-MS/MS according to the methods described in a previous report [9]. Only biotinylated protein spots repeatedly detected using the streptavidin–Alexa Fluor® 488 conjugate were selected for identification. Briefly, the spots were excised from the gels, washed with 50% ACN buffer, dehydrated with 100% ACN, vacuum-dried, and then digested by trypsin. Peptides were extracted with ACN/TFA/ddH_2_O (50∶5:45 v/v/v), and evaporated to complete dryness under a vacuum. The samples were subsequently dissolved in formic acid/ACN/ddH_2_O (0.1∶50:49.9 v/v/v) and analyzed by LC-nanoESI-MS/MS.

MS/MS ion searches were performed on the processed spectra against the 23,677 predicted proteins of *Acropora digitifera* (http://marinegenomics.oist.jp/genomes/downloads?project_id=3; adi_v1.0.1.prot.fa.gz (genome assembly version 1.0)) [17] using the MASCOT search program. First, the 23,677 predicted proteins were annotated by sequence homolog match in NCBI non-redundant protein sequences (nr) database (database releasing date: 2011/06) using BlastP (E value cutoff: 1E^−5^) [18]. For identifying possible functional domains, we conducted RPS-BLAST on Conserved Domain Database (CDD) with predicted proteins [19]. Orthologous assignment and mapping of the predicted proteins to the biological pathways were performed using KEGG Automatic Annotation Server [20]. Secondly, the acquired MS/MS sequences were blasted the annotated proteome of *Acropora digitifera*, as acquired above. The peptide tolerance parameter was 20 ppm, the MS/MS tolerance was 1 Da, and up to one missed cleavage was allowed. Variable modifications were oxidation (M) and carbamidomethyl (C), and fixed modifications were biotin (K) or biotin (N-terminal), or none. The criteria for the positive identification of proteins were set as follows: (i) the MOWSE score against a matched protein was higher than 23 or (ii) the matched protein had the same molecular weight (MW) or p*I* as the SGC biotinylated protein, or (iii) the SGC biotinylated protein aligned significantly to a published cnidarian protein sequence. Possible transmembrane domains of the identified proteins were predicted by TMpred (http://www.ch.embnet.org/software/TMPRED_form.html). Finally, the identified coral proteins blasted NCBInr database with default setting to further identify protein names/species/GI numbers with the highest identity (%) among marine species. Identified proteins were further analyzed by Protein Knowledgebase (UniProtKB) (http://www.uniprot.org/uniprot/) in order to determine their possible functions.

The selected spots on the 2D SDS-PAGE gels were circled, and the spot density was analyzed with ImageMaster (GE Healthcare).

## Results

**Figure 1:**
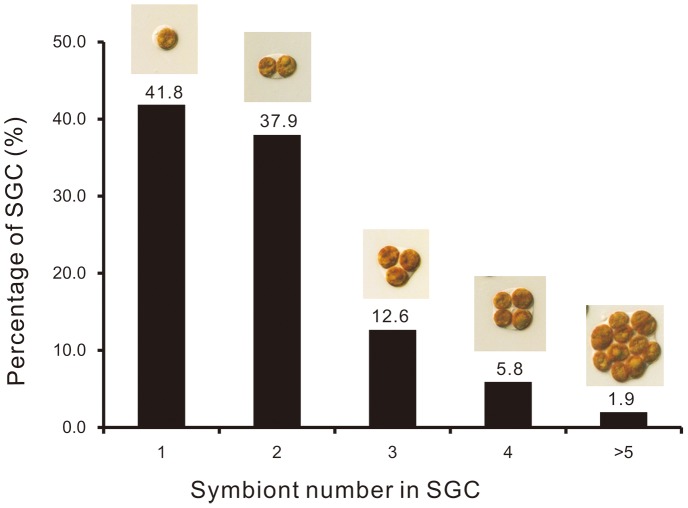
The numeric distribution of *Symbiodinium* within symbiotic gastrodermal cells (SGCs). SGCs were isolated from tentacles of the reef-building coral *Euphyllia glabrescens*, and these host cells (n = 890) were found to contain from one to ten *Symbiodinium*.

We isolated large quantities of homogeneous SGCs from tentacles of the coral *E. glabrescens*. A single SGC typically contained from 1 to 10 endosymbionts (Fig. 1). The majority of them contained either one (41.8%) or two (37.9%) *Symbiodinium* (Fig. 1).

### 1. The Biotinylation of SGC Surfaces

**Figure 2:**
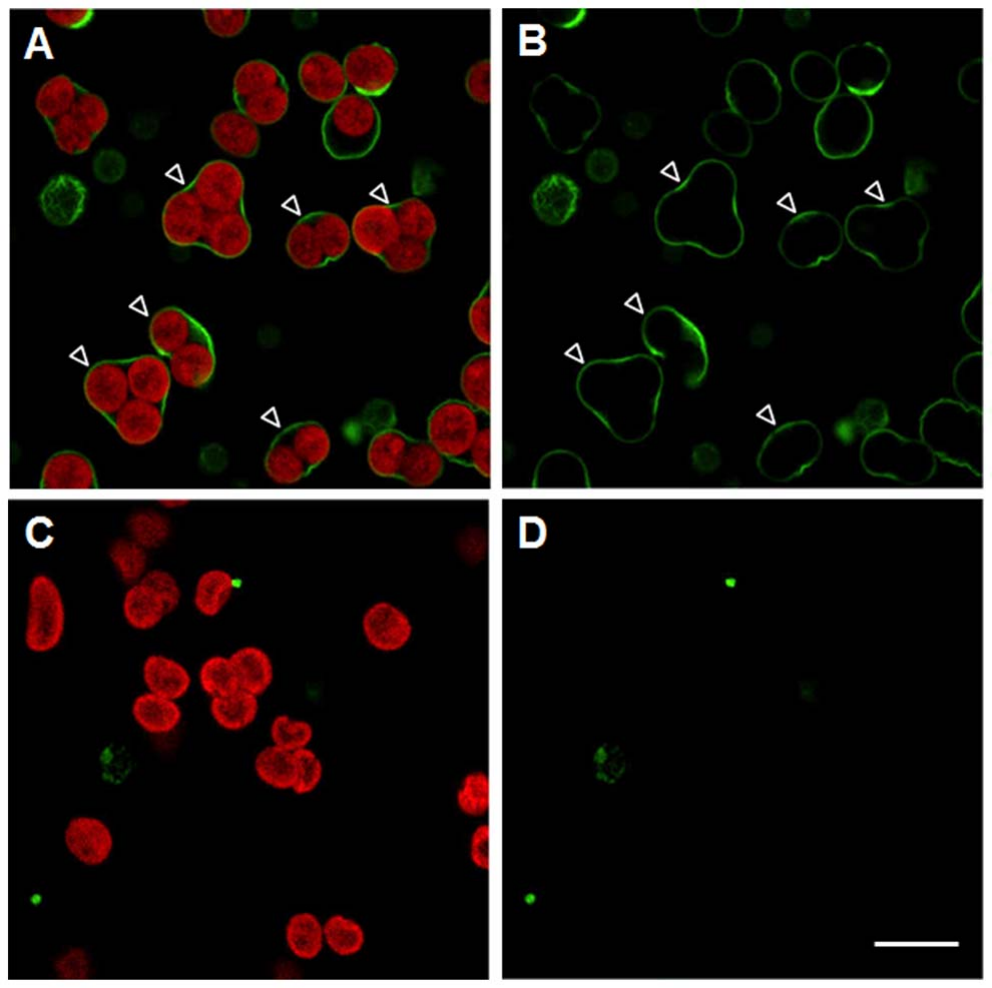
Labeling of symbiotic gastrodermal cell surface proteins by a biotin-streptavidin probe. Biotinylated (A, B) and non-biotinylated (C, D) SGCs were incubated with streptavidin-Alexa Fluor® 488 (green fluorescence) and imaged with a confocal microscope. Fluorescence distribution was examined by confocal microscopy at 543 nm (red fluorescence) in panels A and C and 488 nm (green fluorescence) in all panels. The arrowheads in panels A and B indicate labeling of SGC membranes. Scale bar = 20 µm. The red fluorescence in panels A and represents autofluorescence of *Symbiodinium*.

To investigate the cell surface proteins of SGCs, we used biotin-XX sulfosuccinimidyl ester to chemically conjugate the membrane surface proteins. Biotin-XX sulfosuccinimidyl ester (C_26_H_40_N_5_NaO_10_S_2_, MW 669.74) is a cell-impermeant, amino-reactive agent, which has been widely used to label proteins exposed on the surface of live cells. The biotinylation reaction was performed in amino acid-free ASW, and the sulfosuccinimidyl ester reacts with exposed amino groups of either lysine residues or the N-terminus of surface proteins. Furthermore, as the binding of biotin to streptavidin is one of the strongest non-covalent interactions known (see [9] and references cited therein.), it represents a powerful tool to specifically detect biotinylated proteins using Alexa Fluor® 488 conjugated streptavidin. As shown in Fig. 2, the labeling of fluorescent streptavidin was specific to the surface membranes of biotinylated SGCs (see arrowheads in panels A and B.). In contrast, no fluorescence was observed on the surface of non-biotinylated SGCs (panels C and D).

**Figure 3:**
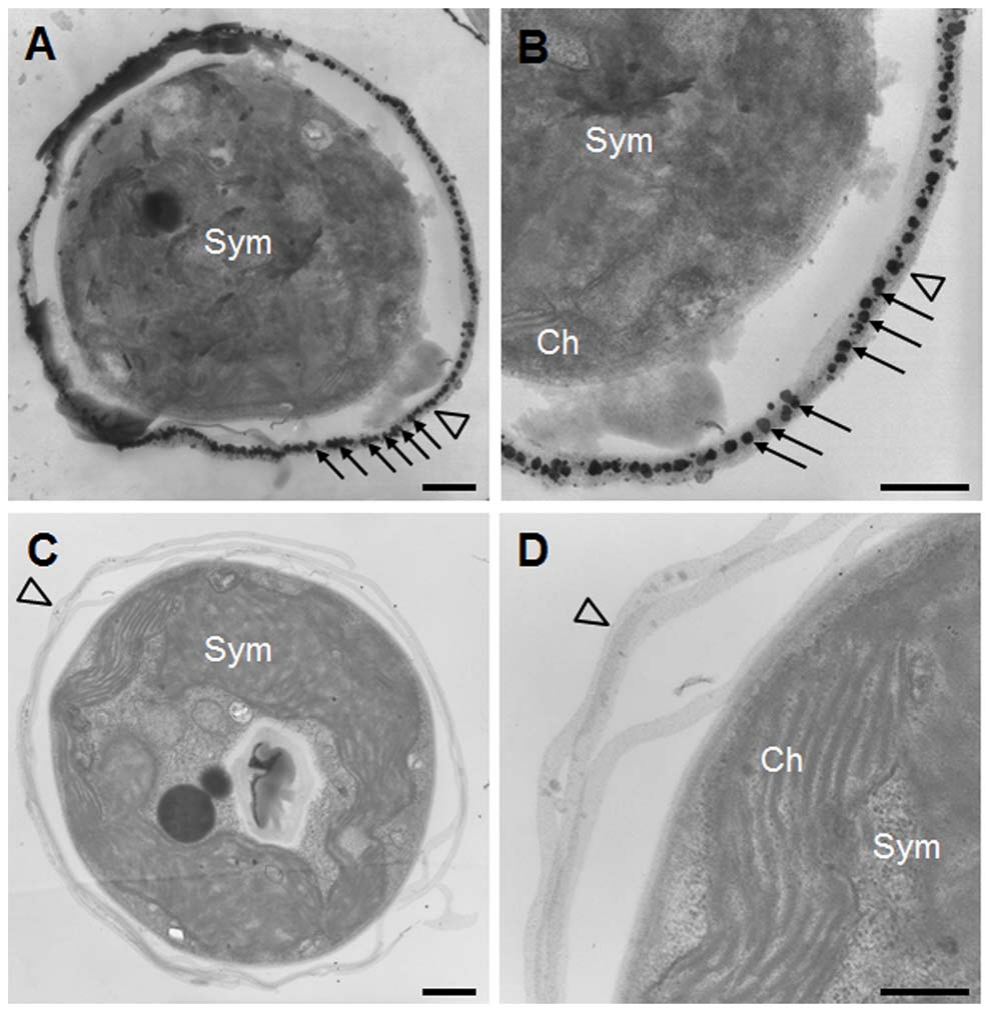
Nanogold-labeling of SGC membranes. The biotinylated (A, B) and non-biotinylated (C, D) SGCs were treated with streptavidin-conjugated nanogold particles, enhanced by silver, and then observed by transmission electron microscopy. Silver enhanced-nanogold particles (see arrows) only appeared on the biotinylated SGC membranes (indicated by arrowheads). Sym: *Symbiodinium*; Ch: chloroplast. Scale bar = 500 nm.

The biotinylation on the SGC surface was further confirmed by TEM. As shown by arrows in Fig. 3A–B, the silver-enhanced nanogold particles appeared only on the membranes of biotinylated SGCs; no nanogold particles could be visualized on the the membrane of non-biotinylated SGCs (Fig. 3C–D). These results demonstrate the successful biotinylation on the surface of SGCs, but not in the cytoplasm or in the *Symbiodinium*.

### 2. Identification of Biotinylated Proteins by 2-D Gel Electrophoresis and LC-MS/MS

**Figure 4:**
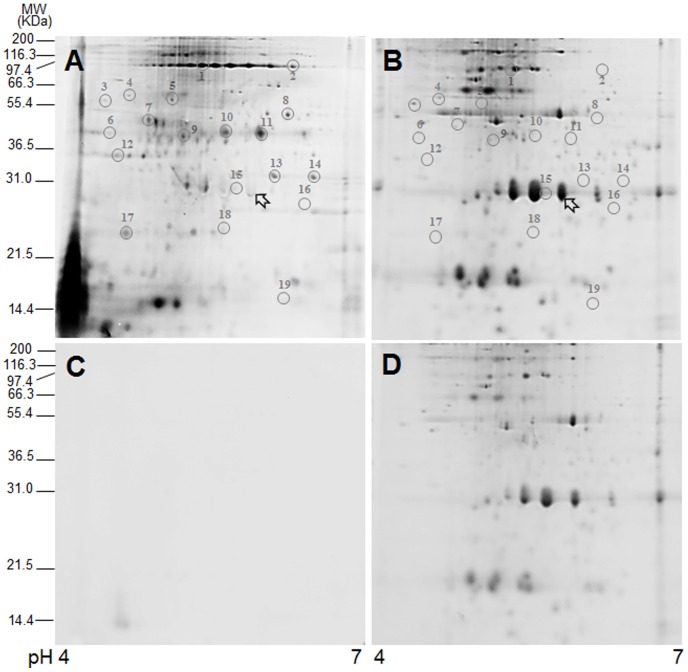
2-dimensional gel electrophoresis of biotinylated SGC proteins. The proteins of biotinylated (A, B) and non-biotinylated (C, D) SGCs were extracted and separated by 2-D gel electrophoresis. The gel was stained with streptavidin-Alexa Fluor® 488 (A, C) first and then SYPRO® Ruby (B, D). The circles in A and B indicate the biotinylated SGC proteins which were successfully identified by LC-MS/MS (see list in Table 1.). The blank arrowheads in A and B indicate the peridinin-chlorophyll a-binding protein (PCP, an intracellular protein of *Symbiodinium*).

Proteins were extracted from biotinylated SGCs and separated by 2-D gel electrophoresis (Fig. 4). Biotinylated proteins in the gel were then detected by streptavidin conjugated with Alexa Fluor® 488 (Fig. 4A). Afterwards, total proteins on the same gel were visualized by SYPRO® Ruby (Fig. 4B). By comparing the total protein profile (Fig. 4B) with that of the biotinylated proteins (Fig. 4A), the specificity of the biotinylation on the cell surface could be validated. For instance, the peridinin-chlorophyll a-binding protein (PCP; an intracellular protein of *Symbiodinium*) was not biotinylated, as indicated by blank arrowheads in Fig. 4A and 4B). This demonstrates the cell impermeability of the biotin-XX sulfosuccinimidyl ester and confirms that only proteins on the membrane surface of SGCs were biotinylated.

In order to further confirm the specificity of surface biotinylation, the protein profile of non-biotinylated SGCs was observed (Fig. 4C–D). As shown in Fig. 4C, there were no protein spots detected with streptavidin-Alexa Fluor® 488 on gels run with proteins extracted from non-biotinylated SGCs. Secondly, most of the biotinylated proteins (Fig. 4A) were not concentrated enough to be identified by SYPRO® Ruby staining (Fig. 4B). This indicates that the surface protein species being biotinylated were limited and furthermore suggests that the detection of biotinylated proteins using streptavidin is sensitive and selective.

A total of 44 biotinylated protein spots were analyzed by liquid chromatography tandem mass spectrometry (LC-MS/MS). Nineteen (19) of them (see the selected protein spots in Fig. 4A.) could be identified according to the criteria described above (Table 1) using a coral protein database. Most identified proteins belonged to three functional categories: molecular chaperones/stress response (37%), cytoskeleton (26%), and energy metabolism (11%).

**Table 1:** Identification of biotinylated SGC surface proteinsa).

Protein name	The matched species/taxonomy in NCBIg)	Spot no.	Identity(%)f)	GI No.	MS/mpsd)	Sequencecoverage(%)e)	PredictedMW/PI	ObservedMW/PI	TM domain(numbers)c)	*Acropora*databaseno.b)	Relativeratio(folds) ofbiotinylated*vs* totalproteinsh)	Reference
**Molecular chaperon**												
Mitochondrial 60 kDaheat shock protein	*Anemonia viridis*(Sea anemone)/Cnidarian	5	82	40647591	252/6(6)	9.6	62.352/5.25	61/5.0	+ (2)	v1.08508	1.63	[33], [34]
Heat shock protein 70	*Pocillopora damicornis*(Cauliflower coral)/Cnidarian	12	90	58865330	40/1(1)	2.2	53.887/5.11	36/4.4	+ (1)	v1.07452	4.57	[34]
Predicted protein(Calreticulin)	*Nematostella vectensis*(Starlet sea anemone)/Cnidarian	3	75	156403953	57/2(2)	4.7	46.683/4.29	58/4.2	-	v1.16781	0.65	
Protein disulfideisomerase (PDI)	*Haliotis discus*(Disc abalone)/Mollusca	4	65	126697420	276/6(2)	3.8	58.541/4.58	86.9/4.5	+ (1)	v1.20922	0.36	
Hypothetical proteinBRAFLDRAFT_264882(endoplasmic reticulumprotein ERp29-like)	*Branchiostoma floridae*(Florida lancelet)/Chordata	15	51	260803445	38/2(2)	10.2	25.550/6.06	30.5/5.8	+ (1)	v1.05402	0.2	
Predicted protein(cyclophilin_WD40)	*Nematostella vectensis*(Starlet sea anemone)/Cnidarian	16	74	156364915	27/2(2)	5.2	54.568/6.63	28.6/6.4	-	v1.07773	0.26	
Predicted protein,partial (hypoxia-inducible factor 1)	*Nematostella vectensis*(Starlet sea anemone)/Cnidarian	17	69	156351477	24/3(1)	10.5	27.786/4.78	24.5/4.6	–	v1.09374	1.41	
**Cytoskeleton**												
Beta-actin	*Euphyllia ancora*(Anchor coral)/Cnidarian	9	99	399886890	265/12(10)	28.5	41.7/5.29	44/5.2	+ (2)	v1.09988	2.61	[29]
Beta-actin	*Euphyllia ancora*(Anchor coral)/Cnidarian	10	98	399886890	93/6(5)	15.4	41.719/5.3	43/5.6	+ (2)	v1.11680	10.56	[29]
Beta-actin	*Euphyllia ancora*(Anchor coral)/Cnidarian	11	98	399886890	170/7(6)	18.1	41.719/5.3	47/6.0	+ (2)	v1.11680	8.09	[29]
Predicted protein(Ras_like_GTPase)	*Nematostella vectensis*(Starlet sea anemone)/Cnidarian	13	88	156379905	47/2(2)	18.1	16.211/5.04	33/6.1	+ (1)	v1.13216	8.06	
PREDICTED: echinodermmicrotubule-associatedprotein-like 6-like	*Pundamilia nyererei*(African cichlids)/Chordata	19	67	548555172	28/1(1)	6.8	14.877/5.7	14.6/6.1	-	v1.03377	1.29	
**Energy metabolism**												
ATP synthasealpha chain	*Mus musculus*(House mouse)/Mammal	8	72	148677499	23/5(4)	9.2	48.508/8.7	54/6.0	-	v1.01221	5.65	[35]
Mevalonate kinase,partial	*Monodelphis domestica*(Gray short-tailedopossum)/Mammal	18	45	150387543	29/19(1)	3.4	28.284/6.35	25.3/5.6	+ (1)	v1.11847	0.61	
**Miscellaneous**												
Mucin-associatedsurface protein(MASP)	*Trypanosoma cruzi*strain CL Brener/Excavata	6	28	71660598	33/5(1)	2.2	53.970/4.11	51.9/4.2	-	v1.13096	1.16	
Predicted protein(Glucosidase II betasubunit-like protein)	*Nematostella vectensis*(Starlet sea anemone)/Cnidarian	14	49	156375483	44/1(1)	3.6	37.297/8.19	33.15/6.5	+ (1)	v1.19038	8.35	
Notch	*Nematostella vectensis*(Starlet sea anemone)/Cnidarian	2	48	363895250	29/2(2)	4.1	101.369/6.53	97.4/6.4	+ (3)	v1.22525	6.57	
PREDICTED:serine/threonine-proteinkinase HT1-like	*Glycine max*(soybean)/Streptophyta	1	35	356570516	24/2(2)	4	97.102/6.37	97.4/5.3	+ (2)	v1.11133	2.17	
Centrosomalprotein 63	*Danio rerio* (Zebrafish)/Chordata	7	34	34784865	29/1(1)	2.1	50.167/5.08	52.25/4.9	-	v1.11742	2.2	

^a)^ MS data were first screened against the *Acropora digitifera* protein library (see the section of “Materials and methods”). Matched coral proteins with MS>23 were then blasted to NCBInr database to identify predicted proteins.

^b)^ The serial no. of proteins in *Acroporal digifera* database (http://marinegenomics.oist.jp/genomes/downloads?project_id=3).

^c)^ Transmembrane domains were predicted by TMpred. (http://www.ch.embnet.org/software/TMPRED_form.html).

^d)^ MOWSE score/number of total matched peptides (numbers of different matched peptides) against the *Acropora digitifera* protein library.

^e)^ The coverage of protein spot peptide sequence among matched *Acropora* protein.

^f)^ The percentage of sequence identity between the identified *Acropora* protein and the closet matched protein in NCBInr database.

^g)^ The species were protein spot to blast NCBInr database using the MASCOT search program, and this column shown the closest matched species.

^h)^ Relative fluorescent ratio (fold) of the Alexa Fluor 488 (i.e. biotinylated) over SYPRO (see the “Materials and methods” section).

## Discussion

The SGC plasma membrane plays pivotal roles in the recognition and phagocytosis of *Symbiodinium* [11], [12]. They also play a major role in the regulation of the stability of these endosymbiotic associations [11]. Unfortunately, there is no specific cellular or molecular marker to identify these cells *in situ* unless they harbor *Symbiodinium*. Furthermore, their purification is difficult, a hindrance that has thwarted previous efforts to elucidate the regulatory mechanisms underlying the coral-*Symbiodinium* endosymbiosis [11]. Herein, we utilized a previously develop tissue dissociation method to collect a high concentration of pure SGCs [11], [13] for characterization of surface proteins.

### 1. Surface Protein Biotinylation in Coral SGCs: Advantages and Limitations

The biotin-XX sulfosuccinimidyl ester is a cell-impermeant agent that reacts with exposed amine group of proteins either at lysine residues or at the N-terminus [21]. Therefore, the degree of biotinylation depends on the number of amine groups on the target, as well as the location of the protein on the SGC plasma membrane [9]. The level of biotinylation in each protein spot could be estimated by the relative fluorescence ratio of Alexa Fluor 488 (Fig. 4A) over SYPRO Ruby fluorescence (Fig. 4B; see also the column of “Relative ratio (folds) of biotinylated vs total proteins” in Table 1). For example, actin (spot no. 10) and Ras-like-GTPase (spot no. 13) had strong fluorescent intensity emerging from binding to streptavidin-Alexa Fluor 488, and low fluorescent intensity emerging from binding to SYPRO Ruby, resulting in relatively high fluorescence intensity ratios of 10.56 and 8.06 respectively. This indicates that the *in situ* distribution of these surface proteins may be more distal, which allowed for more amine groups to be biotinylated. On the contrary, proteins with low (<1) streptavidin-Alexa Fluor 488/SYPRO Ruby ratios, such as calreticulin (spot no. 3) might be located in a more internal, proximal orientation, or even embedded within the membrane, hence masking a portion of the amine groups. Therefore, the *in situ* distribution of these identified proteins on the plasma membrane of SGCs could be hypothesized based on this fluorescence ratio. However, whether the variation in this parameter is more driven by cellular location or amino acid sequence remains to be determined.

### 2. SGC Membrane Surface Proteins and their Possible Roles in Regulation of Coral-dinoflagellate Endosymbiosis

The main goal of the present study was to investigate the molecular characteristics of SGC plasma membranes in order to provide insight into their roles in the regulation of the coral-dinoflagellate endosymbiosis. As a consequence, the MS/MS ion search were first performed using a published database with coral genome (*Acropora digitifera*, see [17]). The matched coral proteins were then blasted NCBI database to finalize the identification (see the procedure described in the “Materials and methods” section). As shown in Table 1, among 44 protein spots, nineteen proteins were identified, and most of them belong to cnidarian proteins. Among the nineteen identified proteins, seven were molecular chaperones, five were actin filaments or associated proteins, and two were involved in energy production (Table 1). Besides, there were five proteins with miscellaneous cellular functions. We surmise that these proteins collectively are involved in (1) protein modifications and membrane dynamics necessary to prepare the plasma membrane for cell-cell interactions (i.e., the molecular chaperones) and (2) regulation of membrane trafficking and phagocytosis by actin filaments. These hypotheses are discussed in greater detail below.

#### 2.1. Multifunctional chaperones: cell-cell recognition and regulation of membrane dynamics

Four proteins involved in protein folding were identified, including heat shock protein (HSP) 60, HSP70, calreticulin and protein disulfide isomerase (PDI). HSPs function as molecular chaperones and respond to a variety of stressors, including temperature changes, cellular energy depletion, osmolarity changes, and toxic substance exposure [22], [23]. During the daytime, hyperoxic stress can characterize certain SGCs due to build-up of high oxygen concentrations stemming from *Symbiodinium* photosynthesis. These stress/chaperone-related proteins are involved with refolding of proteins that are denatured by reactive oxygen species (ROS) and prevention of their aggregation and are thus important for the stability of cnidarian–dinoflagellate endosymbioses [22], [24].

Besides these chaperone functions, the HSP60 proteins on the SGC surface could be involved in *Symbiodinium* recognition and consequent phagocytosis. HSP60 has been reported to specifically bind with lipopolysaccharides [25]. The *Symbiodinium*-host recognition process involves lectin/polysaccharide interactions [25], and HSP60 may therefore aid in the regulation of this interaction. Furthermore, as HSP60 was found to enhance phagocytic activity in U937 cells [23], its presence on the surface of SGC plasma membranes may implicate its role in phagocytosis.

Calreticulin, which was also found on the membrane surface of SGCs, binds oligosaccharides with terminal glucose residues [26] and is involved in the biosynthesis of a variety of molecules such as ion channels, surface receptors, integrins, and transporters [27]. Consequently, calreticulin on the surface of SGCs may also function in the recognition of *Symbiodinium* during the initial stages of the endosymbiosis. In addition, a calreticulin homolog that is involved in Ca^2+^ homeostasis and biomineralization has been found in corals [27], [28]. Therefore, calreticulin on the SGC surface may act to regulate Ca^2+^ concentration, a process that could even be linked to calcification.

**2.2. The role of actins in membrane remodeling and regulation of phagocytic activity^.^** *Symbiodinium* (size ∼8–10 µm) typically occupy the majority of the volume of the host gastrodermal cell in which they reside (Fig. 1). In order for the coral host gastrodermal cell to maintain a normal physiology with such a bulky structure inside its cytoplasm, a unique intracellular architecture is required. Actin filament remodeling at cell surfaces is fundamental to regulating membrane elasticity and cell morphology [29], [30]. The present study identified three actin protein spots, with inferred molecular weights ranging from 44 to 47 kDa and p*Is* from 5.2 to 6.0 (Table 1). Besides their roles in signal transduction and protein biosynthesis, Rho family GTPases have also been shown to regulate the actin cytoskeleton and cell adhesion through specific targets in mammalian cells [31]. As both actin and GTPase were highly biotinylated (see the “Relative ratio (folds) of biotinylated *vs* total proteins” column in Table 1.), they may be involved in the cytoskeleton remodeling that would be necessitated by both phagocytosis and cell division of *Symbiodinium* with the SGC. Indeed, the cytoskeletal architecture must be fundamentally altered during the transition from a SGC housing one *Symbiodinium* cell to one housing multiple endosymbionts (Fig. 1) [32].

### 3. Possible Protein Translocation from the SGC Plasma Membrane to the Symbiosome

In a previous study [11] of SGCs isolated from *E. glabrescens*, active membrane trafficking and metabolism was demonstrated, and these processes were shown to be influenced by irradiation. When a *Symbiodinium* is internalized into the host gastrodermal cell, a symbiosome membrane is formed around the *Symbiodinium*. Studies employing immunofluorescence screening with monoclonal antibodies against extracted anemone proteins have found that symbiosome membranes are multi-layered and derived from both the host and *Symbiodinium* [8]. A proteomic analysis of symbiosome membranes of the sea anemone *Aiptasia pulchella* further revealed that the symbiosome membrane may serve as the interface for interactions between the anthozoan host and *Symbiodinium* [9]. In that study, 17 proteins were identified from purified symbiosome membranes of *A. pulchella*, and these proteins were involved in cell recognition, cytoskeletal remodeling, ATP synthesis/proton homeostasis, transport, the stress responses, and prevention of apoptosis [9]. In comparison with the proteomic results of the present study, there are five proteins present in both membranes: actin, HSP60, HSP70, ATP synthase and PDI (see Table 1 and [9].). This might indicate that some components of the symbiosome membrane are conserved across different anthozoan-*Symbiodinium* endosymbioses.
